# Computational models of neurotransmission at cerebellar synapses unveil the impact on network computation

**DOI:** 10.3389/fncom.2022.1006989

**Published:** 2022-10-28

**Authors:** Stefano Masoli, Martina Francesca Rizza, Marialuisa Tognolina, Francesca Prestori, Egidio D’Angelo

**Affiliations:** ^1^Department of Brain and Behavioral Sciences, University of Pavia, Pavia, Italy; ^2^IRCCS Mondino Foundation, Brain Connectivity Center, Pavia, Italy

**Keywords:** cerebellum, synapses, receptors, computational model, purkinje cell, granule cell

## Abstract

The neuroscientific field benefits from the conjoint evolution of experimental and computational techniques, allowing for the reconstruction and simulation of complex models of neurons and synapses. Chemical synapses are characterized by presynaptic vesicle cycling, neurotransmitter diffusion, and postsynaptic receptor activation, which eventually lead to postsynaptic currents and subsequent membrane potential changes. These mechanisms have been accurately modeled for different synapses and receptor types (AMPA, NMDA, and GABA) of the cerebellar cortical network, allowing simulation of their impact on computation. Of special relevance is short-term synaptic plasticity, which generates spatiotemporal filtering in local microcircuits and controls burst transmission and information flow through the network. Here, we present how data-driven computational models recapitulate the properties of neurotransmission at cerebellar synapses. The simulation of microcircuit models is starting to reveal how diverse synaptic mechanisms shape the spatiotemporal profiles of circuit activity and computation.

## Introduction

Synapses are specialized structures that regulate inter-neuronal communication through a process called neurotransmission. While the foundations of synaptic biophysics have been developed in the last century (Koch, 1984; Maxwell Cowan et al., 2001), the advent of sophisticated single-cell recording techniques, such as patch-clamping and calcium imaging, has recently allowed the characterization of specific properties of several synapses in the mammalian brain, in most cases using rodents as the experimental model (Hickman et al., 2017). These results have revealed a previously undisclosed complexity and variety of mechanisms, requiring a further understanding of their functional implications through advanced computational models. Cellular and synaptic biophysics can be translated into mathematical equations (Koch, 1984; Segev, 1998; Brodland, 2015) to generate models of neurons and networks, thus adding a new dimension to the computational investigation of neurotransmission (D’Angelo and Jirsa, 2022). A relevant case is the cerebellar circuit, the synapses of which exhibit a variety of properties. These include presynaptic release and postsynaptic receptor mechanisms, which generate a large spectrum of postsynaptic currents, membrane depolarization kinetics, and short-term plasticity patterns (D’Angelo et al., 2016; De Schepper et al., 2021). In this review, we compared the neurotransmission mechanisms at different cerebellar synapses and the corresponding computational models that unveil the impact of synaptic diversity on microcircuit computation.

### Experimental data and reconstruction of synaptic models

Experimental techniques used to investigate neurotransmission have evolved considerably over the last century (Sotelo, 2008; VoŽeh, 2015; Cavero et al., 2017; Bentivoglio et al., 2019). The extensive application of 3D morphological reconstructions (Guo et al., 2022), immunochemistry, patch-clamp recordings, calcium imaging, genomics, and proteomics has allowed the creation of atlases, such as the Allen Brain Atlas (Gilbert, 2018), which summarizes the knowledge about brain neurons and circuits along with the distribution of synaptic receptors and molecules, both in rodents and humans. Computational models can integrate this large set of experimental observations to reconstruct a variety of synaptic receptor kinetics (AMPA, NMDA, and GABA) and neurotransmission properties (D’Angelo and Jirsa, 2022) (Box 1).

**BOX 1. Simulation environments.**

Simulation environments are a crucial part of biophysical research, providing tools for modeling and testing new concepts and algorithms (Bower, 2015). To choose the most appropriate environment, it is essential to evaluate the levels of biological organization, availability of experimental data, and requirements for computing power.

At the molecular level, highly sophisticated simulators can solve biochemical networks (CellDesigner) (Funahashi et al., 2008), reaction-diffusion systems (STEPS) (Hepburn et al., 2012; Chen and de Schutter, 2017), protein folding and protein-protein interactions (NAMD) (Sotomayor, 2015; Nowak, 2016), and gene regulatory networks (CYTOSCAPE) (Shannon, 2003; Cline et al., 2007). Although these modeling types are grounded in biological reality, they require large amounts of heterogeneous data to visualize large-scale biomolecular networks.

Large-scale simulations can be used to reproduce many different types of tissue and/or organ mechanisms (Dos Santos et al., 2015). Specifically, neural mass models (Jansen and Rit, 1995; Spiegler et al., 2011) are among the most popular mathematical models of brain activity, as they enable the rapid simulation of large populations of neurons and synapses at a spatial scale compatible with electrophysiological experiments (Schirner et al., 2018; Cakan and Obermayer, 2020). An open-source simulation platform optimized for realistic brain connectivity/geometry and neural mass models, The Virtual Brain (TVB) (Sanz Leon et al., 2013), has recently been developed for whole-brain dynamic simulations combining a large-scale brain network model with neuroimaging data, including population activity like electroencephalogram (EEG) Magnetoencephalography (MEG), highly resolved metabolic/vascular signals like the Functional magnetic resonance imaging (fMRI) (Alahmadi et al., 2015), and measures of neuronal connectivity like in the case of diffusion tensor imaging (DTI) (Palesi et al., 2021).

The most popular single-neuron models are:

(1) Conductance-based models, which, with a variety of ion channels of the Hodgkin–Huxley (HH) type (Hodgkin and Huxley, 1952a,b; Hille, 2001), can characterize the biophysical mechanisms of cells in great detail.

(2) Integrate-and-fire (IF) models, which are widely used to describe spiking and bursting behavior (Stein, 1967; Tuckwell, 1988). The most well-known are NEST (Eppler, 2008; Nowke et al., 2015), Brain2 (Stimberg et al., 2019) and LEMS (Cannon et al., 2014). NEURON (Hines and Carnevale, 2001, 2008; Hines et al., 2009), CORENEURON (Kumbhar et al., 2019), GENESIS (Bower and Beeman, 2007), and Arbor (Akar et al., 2019) are well-known simulation environments that allow biophysically realistic single-neuron reconstruction and simulation.

(3) Spike-response neuron (SRN) models, which combine the biological plausibility of HH-type dynamics and the computational performance of IF neurons to simulate tens of thousands of spiking neurons in real time (Izhikevich, 2003). Numerous simulators can combine single-neuron models to generate spiking microcircuit models using specific modeling workflows.

## Mechanisms and models of synaptic transmission

### Chemical synapses

**FIGURE 1:**
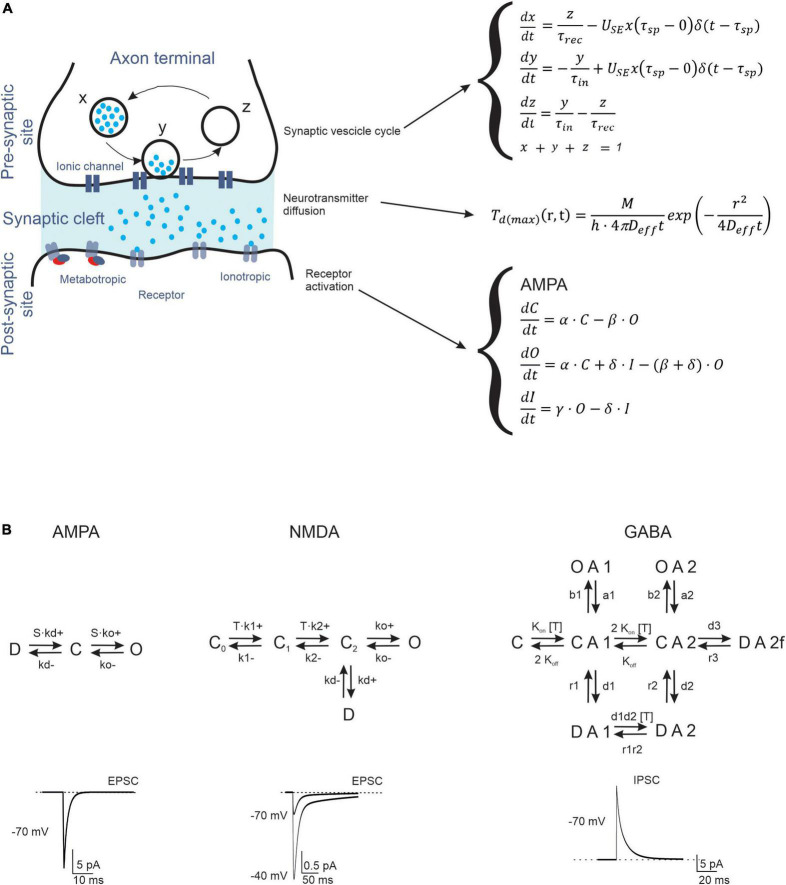
Biophysical representation of synaptic neurotransmission mechanisms. **(A)** Schematic representation of the processes taking place in a synapse (Left). The figure shows a synaptic vesicle in three different states: (X) refilled (immediately available store), (Y) releasing (fusion state) and (Z) depleted (under recovery). Following the release, the neurotransmitter diffuses through the presynaptic cleft and binds to postsynaptic receptors. The differential equations describing the temporal evolution of release, diffusion, and gating (for a generic C = O = D scheme) are indicated to the right. **(B)** The kinetic schemes (Markov chains) describe the transitions of AMPA, NMDA, and GABA receptors through C, D, and O states. These receptors function as ligand-operated channels that can visit multiple states (C: closed, O: open, I: inactive) regulating ionic current flow. Examples of traces are shown at the bottom (the NMDA current has two traces at different holding potentials, as indicated).

A typical chemical synapse requires juxtaposition between the axon of one neuron and the dendrite of another (Südhof and Malenka, 2008). The two sites, called pre- and postsynaptic, do not touch each other, leaving a space called the synaptic cleft. The fusion of vesicles with the presynaptic membrane releases the neurotransmitter, which diffuses to the postsynaptic receptors, where it can open ionic channels (ionotropic receptors) (Andreae and Burrone, 2014; Batool et al., 2019) or interact with G proteins and various enzymes to produce second messengers (metabotropic receptors) (Marcaggi et al., 2009). Thus, the models of neurotransmission should contain a representation of (1) the vesicle cycle, (2) neurotransmitter diffusion, and (3) receptor activation (Figure 1). The properties of some cerebellar synaptic computational models are listed in Table 1.

**TABLE 1:** Properties of cerebellar synapses.

	*U*	τ_facil_	τ_rec_	τ_in_	*Gmax AMPA*	*Subtype*	*Gmax NMDA*	*Subtype*	*GABA-A*	*Subtype*	*REF*
Mf-GrC	0.43	5	8	1	1,200	GluR2	18,800	NR2A-NR2C	−	−	*a*
	0.46	No presynaptic			1,200	GluR2	−	−	−	−	*b*
	0.416	10.8	35.1	3	700	GluR2	−	−	−	−	*c*
	0.416	10.8	35.1	3	−	-	10,000	NR2A-NR2C	−	−	*c*
	0.42	8	5	1	1,200	GluR2	18,800	NR2A-NR2C	−	−	*d*
GoC-GrC	0.35	58	36	1	1,050	GluR2	−	−	−	α1/6	*e*
Mf-GoC	0.43	5	8	1	1,200	GluR2	10,000	NR2B	−	−	*f*
GrC-GoC pf	0.4	54	35.1	1	1,200	GluR2	−	−	−	−	*f*
GrC-GoC aa	0.4	54	35.1	1	1,200	GluR2	10,000	NR2B	−	−	*f*
GrC-GoC (pf-aa)	No presynaptic				2,000	GluR2					*g*
GrC-PC pf	0.13	54	35.1	1	2,800	GluR2	−	−	−	−	*g*
GrC-PC aa	0.13	54	35.1	1	2,800	GluR2	−	−	−	−	*g*
GrC-SC	0.15	10.8	35.1	1	2,300	GluR2	10,000	NR2B	−	−	*h*
GrC-BC	0.15	10.8	35.1	1	2,300	GluR2	10,000	NR2B	−	−	*i*
SC-PC	0.35	4	15	1	−	−	−	−	2,600	α1	*h*
BC-PC	0.35	4	15	1	−	−	−	−	2,600	α1	*i*
SC-SC	0.42	4	38.7	1	−	−	−	−	1,600	α1	*h*
BC-BC	0.42	4	38.7	1	−	−	−	−	1,600	α1	*i*

The properties of the cerebellar circuit synapses have been parametrized by fitting synaptic current trains (Nieus et al., 2006, 2014). The specific models have been reported in references a (Nieus et al., 2006), b (Saftenku, 2005), c (Diwakar et al., 2011), d (Sudhakar et al., 2017), e (Nieus et al., 2014), f (Masoli et al., 2020a), g (Masoli and D’Angelo, 2017), h (Rizza et al., 2021), and i (Rizza et al., 2021), and in unpublished data. In addition, all the parameters to be used in local microcircuits (refs a-f), are now used in the BSB model of the whole cerebellar cortical network (De Schepper et al., 2021). It should be noted that the parallel fiber synapses are strongly facilitating (low U), while all other synapses are depressing (high U) or neutral (depending on the balance between U, ^τ^facil, and ^τ^rec). The NMDA conductance value is higher than AMPA due to low channel opening probability.

#### Presynaptic site—Vesicle cycle

The presynaptic site contains complex biochemical machinery for the production, storage, and release of vesicles that contain neurotransmitters (Figure 1A). To reduce the complexity into a manageable set of equations, the Tsodyks–Markram model was built to account for the main states (X, Y, Z). When a presynaptic spike arrives, the variable X represents the neurotransmitter available for release, Y the amount of neurotransmitter released, and Z the recovered neurotransmitter (Tsodyks et al., 1998). The entire process is controlled by the time constants of recovery of the releasable neurotransmitter (τ_*rec*_), facilitation (τ_*facil*_), and inactivation (τ_*in*_), and the probability of release (U). These parameters effectively regulate short-term synaptic plasticity, including facilitation and depression (D’Angelo et al., 2005). Fitting these models to experimental data allows for the investigation of presynaptic dynamics. The Tsodyks–Markram model has been successfully used in different large-scale networks, such as the neocortex (Markram et al., 2015), hippocampus (Romani et al., 2022), striatum, and cerebellum (Solinas et al., 2010; Sudhakar et al., 2017; Florimbi et al., 2021).

#### Synaptic cleft—Neurotransmitter diffusion

Neurotransmitter homeostasis in and around a synapse involves complex processes, such as diffusion, binding to receptors, and uptake by glial cells (Figure 1A). Various neurotransmitter diffusion-based modeling studies have focused on synaptic receptor activation (Clements et al., 1992; Franks et al., 2002) and evaluated the access of neurotransmitters to synaptic and extra-synaptic locations, considering sequestration by glial cells (Barbour, 2001; Diamond, 2005). These models have been developed and solved using analytical (Kleinle et al., 1996), continuum (Rusakov and Kullmann, 1998; Rusakov, 2001), and stochastic approaches (Franks et al., 2002; Tao and Nicholson, 2004; Savtchenko and Rusakov, 2007; Zheng et al., 2008), maintaining, as a common simplifying feature, the assumption of simple geometrical configurations of the synaptic cleft.

#### Postsynaptic site—Receptor activation and current generation

Markov kinetic models can accurately reproduce electrophysiological and biophysical aspects of different ligand-gated receptors. The simplest Markov model is the Markov chain, which is routinely used to model transitions between states (e.g., closed, open, and inactivated states) (Yamada and Zucker, 1992; Destexhe et al., 1994).

AMPA receptors are heterotetramers composed of two pairs of dimers, in which GluR2 is the main subunit. All subunits (1–4) are distributed throughout the cerebellum: GluR1 and GluR2 are highly expressed in all layers, while GluR3 and GluR4 are detected at a lower concentration in the granular layer (Day et al., 1995; Greger et al., 2007), on Purkinje cell (PC) dendrites, which are required to induce long-term potentiation (LTP) and on Bergmann glia (Saab et al., 2012). The typical receptor of GrCs is composed of GluR2 and GluR4 (DiGregorio et al., 2007) and their participation in expansion recoding and associative memory has been reported (Kita et al., 2021). The most basic AMPA receptor model has three states (open, closed, and desensitized), which can be extended to account for subconductance states (e.g., multiple open, desensitization, and deactivation states), with transitions between states described by Markov models (Koike et al., 2000; Wadiche and Jahr, 2001; DiGregorio et al., 2007; Bennani et al., 2009).

NMDA receptors are heterotetramers composed of two obligatory NR1 and NR2 subunits, which exist as four distinct subtypes (A-D). In general, Markov models for NMDA receptors contain more states compared to those for AMPA receptors, to consider the different biophysical properties conferred by NR2 subunit variability (Figure 1B; Laube et al., 1998; Cull-Candy et al., 2001; Cull-Candy and Leszkiewicz, 2004). Kinetic analysis of postsynaptic currents induced by glutamate during prolonged exposure to glycine has revealed two open states and five shut states (three closed and two desensitized states) (Iacobucci and Popescu, 2018). If we consider the modulation of NMDA receptor desensitization by glycine (Benveniste et al., 1990) or calcium-induced inactivation (Vyklický, 1993), 12 states can be predicted.

GABA_*A*_ receptors are heteropentamers formed by various combinations of subunits: α1-6, β1-4, γ1-4, δ, ε, φ, ρ1-3, and θ (Olsen and Sieghart, 2008). The most common stoichiometry in the mammalian CNS is thought to be two α, two β, and one γ subunits (s) (Baumann et al., 2001, 2002; Olsen and Sieghart, 2008). Although this extensive structural diversity is not completely understood, some subunits confer distinct biophysical and pharmacological properties, especially α subunits, which are thought to play a key role in GABA_*A*_ receptor function. For example, GABA_*A*_ receptors containing α1–3 are intra-synaptic and mediate phasic inhibition, whereas receptors containing α4–6 are largely extra-synaptic and mediate tonic inhibition (Farrant and Nusser, 2005). Several functional studies have shown that gating of the GABA_*A*_ receptor is complex, and there is no consensus kinetic model (Gielen et al., 2012; Szczot et al., 2014; Kisiel et al., 2018). Brief GABA pulses to outside-out macro patches excised from neurons produce macroscopic currents characterized by rapid activation and bi-exponential decay, and desensitization kinetics consistent with long closed states (Maconochie et al., 1994; Jones and Westbrook, 1995). Instead, kinetic analysis of single-channel currents from transiently transfected cells provides simple models for GABA_*A*_ receptor gating containing multiple open and closed components (Lema and Auerbach, 2006).

### Electrical synapses

Electrical synapses are widespread in the mammalian nervous system, including in the human brain. Gap junctions (GJs) mediate electrical synaptic transmission and are composed of two hemichannels (connexons), each of which is formed by integral membrane proteins called connexins (for review see Kumar and Gilula, 1996; Evans and Martin, 2002; Goodenough and Paul, 2009; Nielsen et al., 2012). Emerging evidence indicates that electrical synapses are complex, functionally diverse, and highly modifiable structures that govern the synchrony of rhythmic activity and the timing of spikes in coupled neuronal networks (Destexhe et al., 1998; Chow and Kopell, 2000; Haas and Landisman, 2012; Sevetson and Haas, 2015; Pernelle et al., 2018; Pham and Haas, 2018, 2019). Furthermore, electrical synapses can act as low-pass filters and transfer spikes after hyperpolarization, thereby inducing efficient spike-dependent depression (Vaughn and Haas, 2022).

## The functional richness of the cerebellum

The cerebellum plays a well-established role in motor coordination, and recent studies have established that it also contributes to higher-order cognitive, emotional, and perceptual processing (Schmahmann, 2010; Bastian and Haslam, 2011; D’Angelo and Casali, 2012; van Beugen et al., 2013; Koziol et al., 2014; Baumann et al., 2015; D’Angelo, 2019). Given its extremely regular cytoarchitecture and the distinctive morphological and electrophysiological profiles of neurons and synapses (Hansel et al., 2001; D’Angelo and De Zeeuw, 2009), computational and mathematical approaches are more tractable for the cerebellum than for other brain areas (De Zeeuw et al., 2021). However, recent studies have suggested that cerebellar organization and properties are more complex than initially thought, indicating an impressive diversity of molecular (Tang et al., 2017), cellular (Nedelescu and Abdelhack, 2013; Nedelescu et al., 2018), and circuit mechanisms (Steuber et al., 2007; Engbers et al., 2013; Cerminara et al., 2015; Zhou et al., 2015; Kozareva et al., 2021).

### Realistic multi-compartmental single-cell models

Cerebellar physiology, covering neuronal elements, synaptic connections, and circuit organization, has been intensively studied in the vertebrate brain, allowing the generation of biologically detailed models at multiple levels of complexity (D’Angelo et al., 2016). These models are based on the biophysical and anatomical properties of real neurons, including three-dimensional (3D) spatial morphology, voltage- and ligand-gated ion channels, intracellular calcium dynamics, cell densities, synaptic connectivity patterns, and cytoplasmic processes (Koch, 1998; De Schutter, 2001; D’Angelo and Casali, 2012). The most recent realistic multi-compartmental models of cerebellar neurons and synapses have been developed by integrating knowledge from various sources and capturing a rich variety of neuronal dynamic behaviors during complex neurotransmission patterns (D’Angelo et al., 2001; Solinas et al., 2007a,b; Diwakar et al., 2009; Subramaniyam et al., 2014; Masoli et al., 2015, 2020a,b; Florimbi et al., 2016, 2018, 2021; Masoli and D’Angelo, 2017; Torti et al., 2019; Rizza et al., 2021) (Box 2).

**BOX 2. The NEURON simulator and the brain scaffold builder.**

NEURON is a simulation environment for creating electrical and chemical signaling models of neurons and networks of neurons that are closely related to experimental data (Hines and Carnevale, 2001, 2008; Hines et al., 2009). NEURON was originally designed to facilitate the development of neuronal models, in which complex intrinsic properties and extensive cell configurations play significant roles. This includes facilities for characterizing the longitudinal distribution of ion concentrations and representing the connection network in a computationally efficient manner. The most well-known example of syntax in NEURON is the concept of continuous cable sections (analogous to an unbranched axon), which can be combined to form a tree-shaped structure (Yihe and Timofeeva, 2020). A section can be endowed with membrane properties (including channels, synapses, and ionic concentrations) that change continually with the position along its length. The primary purpose is to determine the physical properties of a neuron without regard for the numerical issue of the size of spatial compartments and, thus, to help the user focus on neuronal anatomy rather than computational details (e.g., cable equation) (Hines et al., 2009).

NEURON programming has been performed using a high-order calculator (HOC), an interpreted language with C-like syntax that has been expanded to include a domain-specific language called the “NEURON Model Description Language” (NMODL) (Hines and Carnevale, 1997, 2001). NMODL allows the incorporation of a wide range of membrane mechanisms such as voltage- and ligand-gated ion channels and active transport, and point processes such as synapses. More recently, Python was adopted as an alternative language, making available within NEURON a very extensive suite of libraries, such as BluePyOpt for ionic conductance (Van Geit et al., 2016) and Netpyne for biological neuronal networks (Dura-Bernal et al., 2019).

The brain scaffold builder (BSB) (De Schepper et al., 2021) is a framework for reconstructing and simulating neuronal network models with single neurons that have been previously reconstructed using NEURON. The scaffold modeling workflow consists of three main phases: configuration, reconstruction, and simulation. The core concepts of the framework during the reconstruction phase are (i) the network volume; (ii) the cell types, which determine the properties of cell populations, such as their spatial representation (soma radius, geometrical extension, and/or morphologies) and density information; (iii) the placement of said cell types into subspaces of the network volume; and (iv) the connectivity between cell types using certain connection strategies. With this information, the framework places and connects cells and allows the simulation of various functional conditions. This last point directly addresses synapses, which are the object of this review.

### The granular layer

**FIGURE 2:**
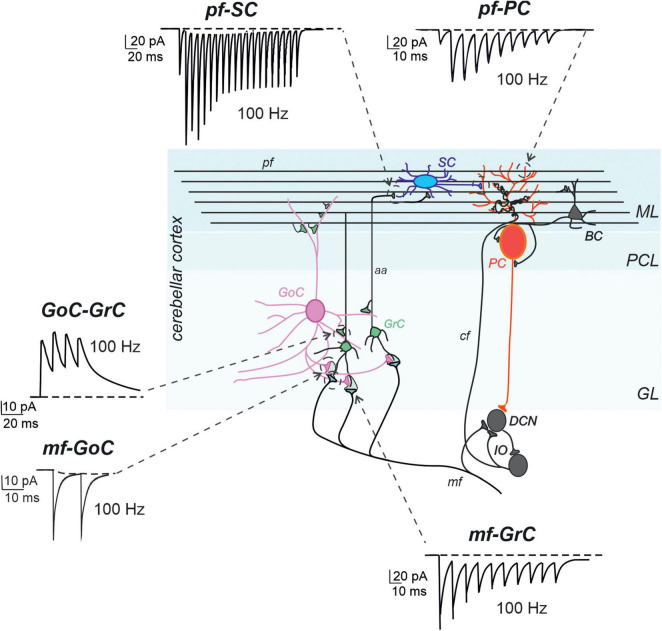
Models of synaptic transmission at cerebellar synapses. Schematic representation of the main elements of the cerebellar circuit along with exemplar simulations of repetitive neurotransmission (EPSC and IPSCs). The cerebellar cortex receives two excitatory inputs: mossy fibers (mf) and climbing fibers (cf). The latter originates in the inferior olive (IO). Both inputs send collaterals to DCN before entering the cerebellar cortex. Mfs contact GrC and GoC. Cfs, contact PC in the purkinje cell layer (PCL). The ascending axons (aa) of the GrCs reach the molecular layer (ML) where it bifurcates into parallel fibers (pf), which synapse onto PC and ML interneurons [stellate cells (SC) and basket cell (BCs)]. The activity of PCs is under inhibitory control by SCs and BCs. The only output of the cortex is provided by PCs, which project to the DCN and provide the main output of the cerebellar circuit. The DCNs project to the IO and in turn to PCs generating a loop mediated by the cfs [modified from D’Angelo et al. (2016)]. The simulated traces are taken from the following papers: mf-GrC, (Nieus et al., 2006), pf-SC (Rizza et al., 2021), GoC-GrC (Nieus et al., 2014), pf-PC (Masoli and D’Angelo, 2017), mf-GoC and GrC-GoC (Masoli et al., 2020a). All the traces reproduce 100 Hz activity. Note the short-term depression at the mf-GrC synapse and short-term facilitation at the pf-PC and pf-SC synapses, while GoC-GrC and mf-GoC demonstrate little temporal dynamics.

The granular layer is the input layer of the cerebellar cortex. It receives mossy fibers, one of the major inputs to the cerebellum, originating from multiple brainstem nuclei, and is composed of different types of cells. The most numerous are small and closely packed granule cells (GrCs), which emit four short dendrites on average and a single axon ascending vertically into the molecular layer, where they bifurcate to form parallel fibers. A few types of interneurons, including excitatory unipolar brush cells (UBCs), inhibitory Golgi cells (GoCs), and Lugaro cells, are located in this layer. GoC axons are profusely arborized and terminate on GrC dendrites, together with mossy fibers, in a peculiar synaptic structure called a “glomerulus” (Figure 2; Mugnaini et al., 1997, 2011; Voogd and Glickstein, 1998).

The properties of mossy fiber-GrC synapses have been the subject of extensive electrophysiological *in vitro* studies (Delvendahl and Hallermann, 2016). Specifically, synaptic currents (EPSCs) have two distinct kinetic components: an early fast AMPA receptor-mediated component and a later longer-lasting NMDA receptor-mediated component (Silver et al., 1992; D’Angelo et al., 1999; DiGregorio et al., 2002; Sola et al., 2004; Nieus et al., 2006; Baade et al., 2016). The NMDA receptor subunit composition undergoes a developmental switch from GluN2A/B to GluN2C-containing receptors (Farrant et al., 1994; Monyer et al., 1994). The GrC spiking output is enhanced in a non-linear manner by membrane depolarization owing to voltage-dependent NMDA receptor activation (D’Angelo et al., 1995; Cathala et al., 2000; Imamura et al., 2000; Rothman et al., 2009; Schwartz et al., 2012). Other factors, such as glutamate spillover, synaptic plasticity, and inhibition (e.g., feed-forward, feedback, and lateral) also affect the integration of GrC information (Mapelli and D’Angelo, 2007; Kanichay and Silver, 2008; Hallermann and Silver, 2013). By combining data collected from electrophysiological experiments and biophysical analyses *in vitro* (D’Angelo et al., 2001; Nieus et al., 2006; Solinas et al., 2007a,b, 2010; Diwakar et al., 2009) and *in vivo* (Kase et al., 1980; Chadderton et al., 2004; Jörntell and Ekerot, 2006; Rancz et al., 2007), large-scale models of the granular layer have been used to predict circuit spatiotemporal coding properties (Maex and De Schutter, 1998; Medina and Mauk, 2000; Solinas et al., 2010; Kalmbach et al., 2011). Interestingly, the realistic computational model of the granular layer network dynamics developed by Solinas et al. (2010) established different roles for the main components of inhibition. Lateral inhibition is crucial for determining center-surface effects [GrCs in the core are more activated than those in the surrounding area (Mapelli and D’Angelo, 2007; D’Angelo and Casali, 2012; Prestori et al., 2019)], feed-forward inhibition to control the temporal window during which GrCs integrate excitatory inputs (D’Angelo and De Zeeuw, 2009; D’Angelo and Casali, 2012; Gandolfi et al., 2014; Eshra et al., 2019; Masoli et al., 2020b), and feedback inhibition to favor coherent oscillations in the theta frequency band (D’Angelo and Casali, 2012; Mapelli et al., 2014).

In contrast to the canonical view describing GrCs as a homogenous population of neurons generating regular firing, our recently published work combining data from simulations and experimental findings on GrC electro responsive properties has identified different subtypes: adapting, non-adapting and accelerating GrCs (Masoli et al., 2020b). Adaptation occurs via Ca^2+^ influx through high-threshold Ca^2+^ conductance and subsequent increase in Ca^2+^-activated K^+^ currents, causing an after-hyperpolarization potential after a burst of spikes (D’Angelo et al., 1998) whereas acceleration is correlated with TRPM4 channels, which are primarily described in PCs (Kim et al., 2009, 2013; Menigoz et al., 2016). Remarkably, our model, derived from previous models (D’Angelo et al., 2001; Nieus et al., 2006; Diwakar et al., 2009) and upgraded to account for sophisticated mechanisms of spike generation, predicted that TRPM4 currents, first recorded in GrCs, coupled to calmodulin through intracellular Ca^2+^ changes, could effectively generate firing acceleration observed experimentally (Masoli et al., 2020b). Remarkably, mossy fiber-GrC synapse simulations showed that fine-tuning of adaptation/acceleration and short-term plasticity generated effective mossy fiber-GrC transmission channels, which could enrich the processing of incoming spike trains and enhance spatiotemporal coding at the cerebellar input stage (Gabbiani et al., 1994), according to the theoretical prediction of the adaptive filter model (Marr, 1969; Dean and Porrill, 2011; Rössert et al., 2015; D’Angelo et al., 2016).

Neurotransmission mechanisms at the mossy fiber-GrC synapse have been rigorously investigated, providing a workbench for synaptic investigations in brain slices (D’Angelo et al., 1990; Silver et al., 1992; Mitchell and Silver, 2003) and accurate neurotransmission models, including spillover (DiGregorio et al., 2002) and single-channel opening properties. The AMPA current transient is ultra-fast and controls the high-fidelity millisecond-precise transmission, while a slow spillover-driven persistent component contributes to regulate bursting. The release probability was set to 0.43 to ensure a marked short-term depression, but these synapses were observed to range from 0.4 to 0.6/0.8 (D’Errico et al., 2009), causing adapting discharges in response to mossy fiber bursts. The release probability increases with LTP and decreases with long-term depression (LTD), tuning the first spike delay over a 100 ms window (Nieus et al., 2006). The NMDA current changes during development (Cathala et al., 2000) and can be activated through spillover and non-linearly boosts GrC spikes, generating burst-burst (detonator) retransmission along the mossy fiber-GrC pathway (D’Angelo et al., 1993, 1995). In combination with these specialized neurotransmission properties, the richness of GrC intrinsic electro-responsiveness can produce a variety of transmission patterns, causing spatiotemporal reconfiguration of incoming patterns in the granular layer (D’Angelo and De Zeeuw, 2009; Eshra et al., 2019; Casali et al., 2020) and determining the adaptive filtering properties of the cerebellum.

Since their discovery (Golgi, 1874), GoCs have been intensively studied, both experimentally and theoretically (Dieudonné, 1998; Maex and De Schutter, 1998; De Schutter et al., 2000; Geurts et al., 2003; Forti et al., 2006; Solinas et al., 2007a,b; Hull and Regehr, 2012; Cesana et al., 2013). In recent years, electrophysiological measurements have revealed some important aspects of the cellular functions of these neurons. In particular, GoCs have a rich and complex variety of intrinsic electro-responsive properties, including pacemaker activity, resonance in the theta band frequency, and phase resetting (Dieudonné, 1998; Forti et al., 2006; Solinas et al., 2007a,b). Subsequently, these properties have been modeled using specific ionic channels, excitatory and inhibitory synapses, and GJ (Solinas et al., 2007a,b). We recently developed detailed multi-compartmental models of GoCs that faithfully capture the large repertoire of findings from electrophysiological experiments (Masoli et al., 2020a). The principal prediction of these models is that the synaptic activation of apical dendrites through parallel fibers causes slow Ca^2+^-dependent depolarizations that trigger and, more importantly, regulate the number of spikes that back-propagate into basal dendrites with concomitant NMDA receptor unblock (when the mossy fiber synapses onto basal dendrites are active). This dendritic processing can be considered a coincidence detector that controls the availability of NMDA currents in basal dendrites, providing the basis for spike-time-dependent plasticity (STDP) anticipated by theory (Garrido et al., 2016).

UBCs form a complex network with mossy fibers (Balmer and Trussell, 2019), GoCs, GrCs, and other UBCs (Mugnaini et al., 1997). A biologically multi-compartmental model reproduces the intrinsic UBC electroresponsiveness, confirming the key role of H- and Ca^2+^ currents (Subramaniyam et al., 2014). The model accurately predicted the generation of late-onset responses following mossy fiber stimulation and its dependence on the intracellular cAMP cascade and the consequent regulation of TRP currents (Locatelli et al., 2013). Synaptic transmission properties are also controlled by AMPA receptors (Lu et al., 2017), spillover (Balmer et al., 2021), and metabotropic receptor regulation of intrinsic excitable mechanisms (Guo et al., 2021).

### The molecular layer

The outer layer of the cerebellar cortex is a molecular layer. This layer contains massive dendritic trees of PCs and parallel fibers, which travel in a parasagittal direction after bifurcating at the end of the GrC ascending axon. The dendritic trees of PCs are penetrated by thousands of parallel fibers, forming glutamatergic synapses on the spines of at least 400 PCs. These neurons are one of the most complex in the CNS and are endowed with several voltage-dependent ionic channels (Akemann and Knöpfel, 2006; Masoli et al., 2015) and numerous proteins that buffer calcium entry (Anwar et al., 2012). The molecular layer also contains two main inhibitory interneuron types, stellate cells (SCs) and basket cells (BCs). BCs are typically located in the lower third of the molecular layer and form GABAergic synapses on PC bodies (Sultan and Bower, 1998), whereas SCs are usually considered to reside in the upper two-thirds of the molecular layer and form GABAergic synapses on PC dendrites. Both BCs and SCs receive glutamatergic input from parallel fibers, as well as GABAergic input from other BCs and SCs (Figure 2; Palay and Chan-Palay, 1974). There is an open discussion regarding the molecular distinction of interneurons into subtypes, and a third class, the candelabrum cell, has recently been discovered (Kozareva et al., 2021; Osorno et al., 2022). Although specific computational roles have been proposed for GoCs, inhibitory interneurons are located in the granular layer (for review see Galliano et al., 2010), suggesting that the functions of MLIs have been confined to the simplistic idea that they provide feed-forward inhibition to PCs (Eccles et al., 1966; Ito, 1984; Mittmann et al., 2005; Santamaria et al., 2007; Bower, 2010).

Following the initial simplified models (Lennon et al., 2014, 2015), recently, the electro-responsive properties and synaptic dynamics of SCs have been characterized *in vitro*, allowing the identification of a set of electrophysiological properties that have been used to generate multi-compartmental SC models (Rizza et al., 2021). Specifically, SCs have pacemaker activity, spike frequency that increases almost linearly with current intensity, sagging inward rectification in response to hyperpolarizing current injection, and rebound excitation and pause following the depolarizing current step at the end of the hyperpolarization. Moreover, SCs showed marked short-term facilitation during repetitive parallel fiber transmission. Because of synaptic filtering combined with intrinsic electro-responsiveness, spikes were generated by the SC model after a lag and only at high frequencies, making SCs operate as delay-high-pass filters (Rizza et al., 2021). Our models, based on detailed morphologies and precise membrane mechanisms, faithfully reproduced the entire set of available experimental data and allowed us to explore the different functional configurations of the parallel fiber-SC-PC circuit. Interestingly, simulations predicted a new role for SCs, which could provide the molecular layer low-pass and band-pass filtering properties recoding parallel fiber bursts and regulating the PC gain in a frequency-dependent manner (Rizza et al., 2021). These findings expand the adaptive filter view of cerebellar circuit functions (Dean and Porrill, 2011).

### Metabotropic receptor

Metabotropic receptors, rather than generating postsynaptic currents, exert a relatively slow effect on ionic channels, since various enzymes transduce the receptor signal through second messengers and cytoplasmic cascades. This multistep process is critical for fine-tuning slow responses, development, and cellular survival (Yamasaki et al., 2021). In the cerebellum, all mGluR subunits are expressed (except for mGluR3 and mGluR6). The most common is mGluR1, which is prominent in PCs but is also expressed in other cerebellar neurons. Some subtypes of GoC are endowed with mGluR2 and mGluR5, while mGluR4 is restricted to parallel fibers (Knöpfel and Grandes, 2002). This receptor mediates slow electrical responses through TRPC channels that, in the case of PCs, are critical for the development and maintenance of the dendritic structure (Wu and Kapfhammer, 2022).

The GABA(B) receptor can be found in specific locations in the cerebellum (Kulik et al., 2002), especially in PC spines and dendritic shafts, and parallel fibers (Luján et al., 2018). The receptor can cluster with Kir3.x ionic channels and the main PC calcium channel Cav2.1. The presence of both GABA(B) and NMDA receptors in PCs has been suggested to support bistability (Sanders et al., 2013) induced by the activation of climbing fibers (Loewenstein et al., 2005). GABA(B) receptors are also expressed in GrCs, where they modulate the input-output relationship (Mapelli et al., 2014; Bassetti, 2022). A computational model of GABA(B) and Kir3 ionic channels has been built to reproduce physiological responses (Destexhe et al., 1994).

### Electrical synapses and ephaptic coupling

GoCs are electrically coupled via GJs, which enhance low-frequency oscillatory synchronization and resonance in GoC networks (Dugué et al., 2009). Asynchronous excitatory inputs to synchronized GoCs cause rapid and considerable desynchronization (Vervaeke et al., 2010). Few studies, combining experiments and modeling, have investigated how GJ coupling affects the GoC and granular layer oscillation using isolated GoC networks (Dugué et al., 2009; Vervaeke et al., 2010) or including the parallel fiber feedback loop. A more realistic computational model with GoCs synaptically connected in the granular layer (Simões de Souza and De Schutter, 2011) confirmed previous findings (Dugué et al., 2009; Vervaeke et al., 2010), suggesting that GJs between GoCs increased the power of cerebellar cortex oscillations, which were synaptically driven by the feedback loop between GoCs and GrCs. These results suggest a novel function for GJs between GoCs in determining a change in the timing of oscillatory cycles, which subsequently improves GoC synchronization (Simões de Souza and De Schutter, 2011). GoC-GoC GJs have recently been introduced into a model of the cerebellar cortex (De Schepper et al., 2021).

GJs are electrical synapses that have advantages over chemical synapses, such as being instantaneous and having no transmission failures (Hoehne et al., 2020). They can transmit signals in both directions; however, through synaptic rectification, they can transmit signals in a preferred direction only (Snipas et al., 2017). In the cerebellum, SCs form GJs with just another SC to form couples (Alcami and Marty, 2013), while BCs tend to form GJs with multiple neighboring BCs (Kim et al., 2014). The physiological consequences of this organization can be monitored using the different responses elicited by PCs. SCs form synapses on the vast dendritic tree of PCs and have a modulatory effect on spontaneous firing, generating pauses that reach a maximum of 60–70 ms (Steuber et al., 2007). BCs form GABAergic synapses on the soma of the PC and ephaptic connections on the axon initial segment (AIS) (Blot and Barbour, 2014). A PC is in contact with up to seven BC axons, but only one or two of them form functional synapses (Kim et al., 2014). The consequences of this synaptic organization propagate to the deep cerebellar nuclei (Chu et al., 2012).

Ephaptic coupling, a mechanism by which neurons communicate via electrical signals through the extracellular space, was first observed between spontaneously active fibers in mouse spinal nerve roots (Rasminsky, 1980). Shortly after, ephaptic signaling was shown to control synchronization and time-spiking in hippocampal neurons (Taylor and Dudek, 1982). More recently, distinctive characteristics of ephaptic interactions have been observed in both cerebral and cerebellar cells (Anastassiou et al., 2011, 2015; Blot and Barbour, 2014; Han et al., 2018). In the cerebellum, ephaptic coupling has been shown to occur between the axon terminal of BCs (pinceau) and the PC somata (Blot and Barbour, 2014), and between PCs near the AISs (Bender and Trussell, 2012; Han et al., 2018). Ephaptic coupling between PCs differs from that provided by BC synapses onto PCs. In the latter case, ephaptic transmission has an inhibitory effect. The extracellular field produced by the BC pinceau is characterized by two current components, a capacitive current and an ionic current through voltage-activated potassium conductances (the pinceau is devoid of sodium channels) (Laube et al., 1996; Southan and Robertson, 2000; Bobik et al., 2004) resulting in a rapid inhibition of PC firing. In contrast, for PC AISs, the extracellular field is dominated by sodium current flow, producing a large excitatory signal and subsequently promoting the synchronization of nearby PCs. These examples reveal the importance of the distribution of ionic channels within a cell in determining the strength and sign of ephaptic coupling. To date, no study has reproduced these effects on PCs using computational models.

## A summary view

The most critical aspects of synaptic transmission in the cerebellar cortical circuit can be summarized by the parameters of the vesicle release cycle, neurotransmitter diffusion, and postsynaptic receptor activation (Figure 1 and Table 1). A comparative analysis of these parameters, which were extracted by fitting EPSC and IPSC trains at the most important cerebellar cortical synapses, revealed that excitatory synapses formed by ascending axons and parallel fibers emitted by GrCs usually had a lower release probability than others in the circuit. This implies that these synapses mostly show short-term facilitation, while the others show short-term depression. Therefore, the activation of molecular layer interneurons (MLIs) and PCs is subjected to heavy filtering, so that single spikes in mossy fibers do not have remarkable effects, while bursts emitted by GrCs can pass through. This single-synapse filtering is integrated at the cell level by intrinsic excitability and converted into effective circuit filtering by the network neuronal chains. A clear example is that of the MLI-PC sub-circuit in the molecular layer: parallel fiber-MLI synapses facilitate the generation of a high-pass filter in SCs, which then inhibits PCs, causing low-pass filtering of parallel fiber-PC transmissions (Rizza et al., 2021). In the granular layer, the high mossy fiber-GrC release probability combines spillover and non-linear NMDA receptor activation, generating a high-pass filter controlling the phase and composition of burst emission by GrCs. This circuit is further regulated by the inhibitory synapses of the GoCs.

These observations imply that burst-burst retransmission is at the core of the cerebellar coding scheme. Bursts in mossy fibers are initially transformed and retransmitted in the granular layer and are then used to tune the firing frequency of PCs in the molecular layer circuit. Moreover, input bursts prime impulsive or repetitive burst-pause patterns in PCs, SCs, and GoCs, sustained by delays in the feed-forward and feedback circuit inhibitory loops and amplified by the intrinsic electroresponsive properties of neurons. In this system, specific synapse parameters play a key role in tuning transmission and filtering properties.

Another critical factor is neurotransmitter spillover, which sustains bursting at the granular and molecular layer synapses. Moreover, the properties of specific AMPA receptor subtypes (gating kinetics and desensitization) determine the precision of spike emission, whereas the properties of NMDA receptor subunits (regulating gating kinetics, desensitization, and voltage-dependent Mg block) determine the intensity and duration of bursts. Another dimension is introduced by cell-to-cell variability, with differentiated excitable and neurotransmission properties even within the same class of neurons, [e.g., GrCs (Chabrol et al., 2015; Gilmer and Person, 2017; Masoli et al., 2020b; Straub et al., 2020), MLIs (Rizza et al., 2021), and candelabrum cells (Osorno et al., 2022), although not included in this work yet]. Finally, the strategic location of NMDA receptors at certain synapses (mossy fiber-GrC, mossy fiber-GoC, parallel fiber-PC, parallel fiber-MLI), as well as Ca permeation in certain types of AMPA receptors and voltage-gated calcium channels, causes various forms of plasticity that can further tune postsynaptic receptor conductance and presynaptic release probability, regulating the filtering properties of the circuit and local computations.

## Conclusion and future challenges

In conclusion, the properties of neurotransmission and intrinsic electroresponsiveness determined experimentally in the cerebellum have been translated into accurate biophysical models, which, in turn, provide a new view of how the cellular and subcellular organization of neurons and synapses contribute to function. Indeed, while a bewildering number of molecular properties await functional explanation and system integration, simulations of detailed computational models can suggest new directions for experimental investigations and provide hints for understanding neuropathologies (D’Angelo and Jirsa, 2022). For example, alterations in synaptic activity in brain diseases affecting the cerebellum, such as ataxia, dystonia, autism, and paroxysmal kinesigenic dyskinesia (LeDoux et al., 1998; Rinaldo and Hansel, 2010; Brown and Loew, 2012; Won et al., 2013; Power and Empson, 2014; Zhang et al., 2015; Zhang and Sudhof, 2016; White and Sillitoe, 2017; Soda et al., 2019; Binda et al., 2021), remain largely to be elucidated; therefore, their translation into models would require investigation of structural changes, receptors, and biochemical pathways. This entails extending the models to include the mechanisms of metabotropic receptor-dependent neuromodulation and calcium-dependent long-term synaptic plasticity (either in the form of LTP, LTD, or STDP). This, in turn, implies updating and expanding the simulation environment, for example, incorporating a voxelized extracellular space into the simulators, as was recently done in NEURON with the reaction-diffusion module (RxD) (Newton et al., 2018). This module can be used to simulate complex biochemical pathways and diffuse substances into a 3D space. This approach allows for the reconstruction of more detailed synapses with substance diffusion between neurons. Unavoidably, this will bring about an increased computational cost, promoting the development of new modeling paradigms and simulation environments (Chen et al., 2022), implying that brain modeling will depend increasingly on the intersection of biology, computation, and informatics.

Here, we indicated that specific properties of neurotransmission and intrinsic electroresponsiveness shape the spatiotemporal profiles of cerebellar cortical circuit activity, as predicted by motor learning theory and adaptive filter theory (Marr, 1969; Dean and Porrill, 2011; Rössert et al., 2015; D’Angelo et al., 2016). In addition to improving single-neuron and synaptic models, the challenge is threefold. First, the remaining neurons and synapses of the cerebellar network, including unipolar brush, Lugaro, and candelabrum cells (Guo et al., 2021; Miyazaki et al., 2021; Osorno et al., 2022), tune cerebellar circuit activity through recurrent inhibitory loops. Second, these new cellular models and synapses are included in an updated version of the cerebellar cortical model (De Schepper et al., 2021) and then extended to the mesoscale by including the deep cerebellar nuclei (Uusisaari et al., 2007; Uusisaari and Knöpfel, 2011) and inferior olives (Torben-Nielsen et al., 2012). Third, we simulated dynamic activity in the mesoscale network models. Simulations should be paralleled by experimental investigation of synaptic and cellular properties to better understand signal integration and the ensuing dynamics, providing essential information for model construction and validation. The existing models contain experimental data available at the time of construction, so they can be expanded with newer information as they become available. At the same time, missing cerebellar models, such as candelabrum cells (Osorno et al., 2022), have recently seen a surge in interest that can induce other labs to further study them. The missing data for both known and less known neurons can be optimally obtained using advanced recording techniques, including high-density Multi electrode array (Buccino et al., 2022), voltage-sensitive dye imaging and multispot 2-photon laser microscopy (Ebner and Chen, 1995; Gandolfi et al., 2014; Miccoli et al., 2019; Gagliano et al., 2022), as well as tools to target and dissect specific circuit components, such as genetic engineering and optogenetics (Yang et al., 2018; Adam et al., 2019; Tognolina et al., 2022). We envisage that this roadmap will eventually shed new light on the computational implications of synaptic mechanisms and contribute to explaining the intrinsic algorithms of the cerebellar circuit.

## Author contributions

SM, MR, MT, and FP organized and wrote the manuscript and prepared the figures. ED’A revised and contributed to the final version of the manuscript. All authors contributed to the article and approved the submitted version.
